# Liver injury in COVID-19: pathological findings

**DOI:** 10.11604/pamj.2022.41.56.31114

**Published:** 2022-01-20

**Authors:** Mouna Zghal, Marwa Bouhamed, Manel Mellouli, Meriam Triki, Rim Kallel, Lobna Ayedi, Tahya Sellami Boudawara, Saadia Makni

**Affiliations:** 1 Department of Pathology, Habib Bourguiba University Hospital, El Ain, Sfax, Tunisia

**Keywords:** Liver, COVID-19, injury, pathology

## Abstract

Hepatic injuries have been reported in patients with Coronavirus disease 2019 infection, particularly in those with moderate to severe illness. To date, pathological changes caused by severe acute respiratory syndrome coronavirus 2 (SARS-CoV-2) in liver tissue are unclear. Moreover, the mechanisms involved in liver injury in Coronavirus disease 2019 infection are not yet established. In this paper, we summarize the spectrum of pathologic findings of liver injury in patients infected by SARS-CoV-2 and we discuss the clinicopathological correlation and the mechanisms of liver damage in Coronavirus disease 2019 infection.

## Commentary

Coronavirus disease 2019 (COVID-19) caused by severe acute respiratory syndrome coronavirus 2 (SARS-CoV-2) is a current pandemic declared by the World Health Organization (WHO) on March 11^th^, 2020 [1]. COVID-19 has variable clinical presentations. Although pulmonary disease is the primary clinical manifestation of SARS-CoV-2 infection, multiple organ systems can be involved [2]. Current research is focusing on the liver as a site of ongoing SARS-CoV-2 infection. Liver damage in patients with COVID-19 is increasingly being reported, particularly in those with severe diseases [3]. The etiological mechanisms for COVID-19 hepatic injury is multifactorial and still unclear [4]. In this paper, we summarize the spectrum of pathologic findings of liver injury in COVID-19. Moreover, this review describes the potential mechanisms of liver injury in COVID-19, as well as clinicopathologic correlation. PubMed was searched to select relevant literature related to this study.

**Liver pathology and COVID-19:** morphological studies concerning the interpretation of pathological hepatic alterations related to COVID-19 infection are lacking, and most are post-mortem autopsies. Xu *et al*. [5] performed the first post-mortem autopsy specimen. It was a man aged 50-year-old, who succumbed to severe COVID-19 after acute respiratory distress syndrome (ARDS). In this study, the liver histology showed a moderate degree of microvesicular steatosis and both mild lobular and portal activity. The injury could have been caused by either SARS-CoV-2 infection or drug-induced liver injury. In addition, peripheral blood showed significantly reduced but hyper-reactive CD4 and CD8 cells. The levels of proinflammatory CCR6+Th17 in CD4 T cells and cytotoxic granulations in CD8 cells were increased. This redueced CD4 and CD8 cells may also contribute to hepatocyte dysfunction. Liu *et al*. [6] autopsied a COVID-19 patient referred to the hospital for multiple cerebral infarctions. They demonstrate in their study various hepatic lesions such as focal lobular necrosis, lobular lymphocytic and monocytic infiltration, ballooning degeneration of liver cells, and sinusoidal congestion with microthrombosis. Tiam *et al*. [7] performed postmortem liver biopsies of four patients, aged 59 to 81 years (three males and one female), who died of COVID-19 pneumonia. They each had at least one underlying disease, such as cirrhosis, hypertension, diabetes, chronic lymphocytic leukemia, and renal transplantation. The biopsies showed mild centrilobular sinusoidal dilatation as a common nonspecific change of hepatic sections. Mild lobular lymphocytic infiltration was noted in two cases. Focal macrovesicular steatosis with glycogenated nuclei was present in one case. Patchy hepatic necrosis in the periportal and centrilobular areas was observed in one case. No significant lymphocytic infiltration of the portal tracts was seen. Liver tissue from one case revealed regenerative nodules and thick fibrous bands, consistent with his history of cirrhosis. SARS-CoV-2 Ribonucleic acid (RNA) was isolated from liver tissue through reverse transcriptase-polymerase chain reaction (RT-PCR) in one of these patients.

Ji *et al*. [8] examined the liver dysfunction of two hundred and two consecutive patients with confirmed COVID-19. They compared the clinical findings between patients with non-alcoholic fatty liver diseases (NAFLD) history and those without NAFLD. Patients with NAFLD had a higher risk of disease progression (p <0.0001) and longer viral shedding time (p <0.0001). The post-mortem hepatic biopsy in one of these patients showed microvesicular steatosis with overactivation of T cells, suggested collateral liver damage from viral-induced cytotoxic T-cells. Sonzogni and associated [9] reported a study of 48 post-mortem liver biopsies from COVID-19 positive patients. No significant clinical complain of liver disease were seen during hospitalization. The liver samples revealed many vascular alterations: luminal ectasia, thrombosis of portal and sinusoidal vessels, fibrosis of vascular wall and an abnormal configuration of intrahepatic blood vessels characterized by portal vein braches number increase. Minimal inflammation findings were seen. These findings suggest that coagulation dysfunction or endothelial damage could be the main causes of the pathogenesis in COVID-19 liver-related damage. Wang Y *et al*. [4] released a study of 156 patients diagnosed of COVID-19. Sixty-four patients (41%) had elevated aminotransferases. This group of patients had a greater proportion of cough, higher radiology scores and more severe disease (p = 0.007). Four of 156 patients died; three had liver enzyme abnormality. Postmortem liver biopsies were obtained from two cases with abnormal liver transaminases. The pathological findings were similar in these two cases and showed: massive hepatic apoptosis, some binuclear hepatocytes, and moderate steatosis. Other histological features included mild to moderate lobular inflammation and the portal tract with infiltration of predominant lymphocytes and rare neutrophils. Immunohistochemical analyses revealed increased CD68+ cells in hepatic sinusoids consisting of Kupffer cells activation. Scanty CD4+ and CD8+ cells were observed. In Transmission Electron Microscope (TEM), typical Coronavirus particles were found in cytoplasm of hepatocytes. The virions were round, with a size range between 60-120nm, and spike-like shape, without membrane-bound vesicles. Few virions were fragmented. Mitochondrial swelling, obscure cristae, canalicular impairment, dense materials were observed in SARS-CoV-2 infected hepatocytes, which suggest a cytopathic effect caused by SARS-CoV-2 infection.

Wang S and associates [2] performed postmortem percutaneous biopsies of multiple organs from 3 patients who succumbed to COVID-19 pneumonia. One of these three patients presented a moderate increase in serum aminotransferase and a mild increase in total bilirubin. Liver histological features showed coagulative necrosis in centrilobular areas, microvesicular steatosis, few apoptotic hepatocytes, canalicular cholestasis, and inflammatory cell infiltration around the central vein. In-situ hybridisation (ISH) did not detect the SARS-CoV-2 RNAs in hepatocytes or cholangiocytes. According to these findings hepatocyte necrosis and lobular hepatitis might be caused by hepatic ischemia or drug-induced hepatic injury. Fiel *et al*. [3] reported a study of two patients with severe acute hepatitis with high aminotransferases. They were found to be COVID-19 positive via nasopharyngeal swab testing. One of these patients has a history of liver transplantation (LT) for hepatitis C and alcohol-related liver disease. No respiratory manifestations were seen. They underwent liver biopsy that showed acute hepatitis: mixed inflammatory infiltrate composed of lymphocytes, few plasma cells, scattered eosinophils and neutrophils in the portal tract, prominent bile duct damage in the interlobular area, foci of necrosis in centrilobular area, apoptotic hepatocytes, and endotheliitis. Immunohistochemistry for CD61 was positive in only one case, indicative of the presence of fibrin thrombi in terminal hepatic venules. RNAScope for SARS-CoV-2 was positive in the two cases showing dot-like particles in the cytoplasm of infected cells. The diagnosis of acute cellular rejection was retained for patient with Liver transplantation (LT). Wichmann *et al*. [10] conducted autopsies on 12 patients dying of COVID-19. In all cases, the cause of death was found within the lungs or the pulmonary vascular system. The macroscopic examination of the liver showed: hepatomegaly, chronic congestion, fatty change, and shock liver. Reverse transcriptase-polymerase chain reaction (RT-PCR) detected SARS-CoV-2 RNA in the lung in all patients, and five of 12 patients demonstrated high viral RNA titers in the liver, kidney, and heart. Rapkiewciz and associates [1] reported a series of seven COVID-19 autopsies (four female). Thrombosis was the prominent finding in all organs. Liver samples revealed macrovesicular steatosis in all cases, without significant inflammation. Many platelet-fibrin microthrombi appeared in hepatic sinusoids in six cases. Hepatic ischemic necrosis was associated in 2 cases. Hepatic vein thrombus was noted in one case. This finding suggested that thrombosis plays a role in COVID-19 pathogenesis.

**Possible causes of liver damage among COVID-19 patients:** in the current pandemic, hepatic dysfunction is commonly reported in 14-53% of patients with COVID-19, especially in severe cases [3]. The most additional clinical feature in these patients is liver enzyme abnormality. Liver test abnormalities are significantly higher in patients with severe COVID-19 infection and are associated with poor outcomes [4]. In fact, liver involvement may be classified into two types: patients with mild liver test abnormality which is mostly transient, and patients with higher levels of the liver enzyme [8]. Some studies proved the predominant elevation of serum transferase in severe COVID-19 infection [4,5]. Hepatic involvement due to SARS-CoV-2 is unclear. Liver damage appears to be of multifactorial origin [4]. We describe the possible causes of liver damage below.

**Direct cytopathic damage to the virus:** recent data reported that SARS-CoV-2 can affect the liver cell by directly binding to angiotensin-converting enzyme 2 receptor (ACE2), which is expressed in the liver (2.6 %) and bile duct cells (59.7%) [7]. Typical Coronavirus particles had been identified in the cytoplasm of hepatocytes [3,4,7,10]. However, viral inclusions were not identified in the liver tissue in other studies [2]. In addition, histological features such as apoptotic hepatocytes, binuclear cells, reduced CD4+ and CD8+ lymphocytes cells, swelling mitochondria, canalicular impairment suggest viral cytopathic effect [3,4]. Microvesicular stenosis, lobular and portal activity may also be caused by direct SARS-CoV-2 infection [3-5].

**Drug hepatotoxicity:** liver which is primarily the body´s detoxifier influenced by several drugs used in the treatment of COVID-19 [3]. Some studies reported increases in liver enzymes after using antiviral treatment [3]. Yet, this association was not significant in others models [4]. Histologically, microvesicular steatosis, lobular and portal activity and hepatocyte necrosis may have resulted in drug damage [5]. But these findings are not specific [4]. To date, the role of drug-induced liver injury in COVID-19 is unclear.

**Severe inflammatory response:** systemic inflammatory response syndrome may be associated with hepatic injury in patients with severe COVID-19 infection [3]. Cytokine storms appear to be higher in patients with liver dysfunction than those with normal hepatic tests [9]. Patients with severe COVID-19 infection showed over activation of cytotoxic T cells manifested by high Th17 and marked activation of immune-mediated inflammation including C-reactive protein and cytokines (TNF-α, IL-6, and IL-1β) [5,8].

**Other causes of hepatic damage:** hypoxia, acute respiratory distress syndrome (ARDS), and cardiac failure in severe affected COVID-19 cases can predispose to hepatic ischemia resulted in hepatocyte necrosis and lobular hepatitis [2]. However, this cause alone cannot explain hepatic failure, given the presence of liver test abnormality in stable patients [8]. In addition, the presence of hepatic sinusoids and vessels thrombosis explain the role of coagulate dysfunction in liver damage [9]. The pre-existing chronic hepatic disease may predispose patients with COVID-19 to secondary injury [7]. Patients with chronic viral hepatitis and NAFLD disease, are more vulnerable to SARS-CoV-2 infection [8]. Yet, chronic viral hepatitis does not appear to increase the risk of liver injury in COVID-19 in other studies [4]. To date, the impact of pre-existing liver diseases in COVID-19 infection is still lacking.

## Conclusion

COVID-19 caused by SARS-CoV-2 is currently a pandemic which causes frequently pneumonia. Different degrees of liver injury have been reported in patients with COVID-19 infection. Lobular and sinusoidal lymphocytic infiltration, mild steatosis and hepatic necrosis are the main histopathological abnormalities. More significant findings are thrombosis of hepatic sinusoids and vessels without significant inflammation. Bile duct damage is nevertheless rarely reported. Possible mechanisms of liver injury are complex and include direct viral attack, potential hepatotoxicity from therapeutic drug, and COVID-19 hyperinflammatory response. In addition, hypoxia and coagulative dysfunction may also contribute to liver damage. Hepatic dysfunctions may differ in individual patients. Although temporal derangement of hepatic parameters are depicted, liver injury may be associated with poor outcome, particularly in severe forms and in patients with pre-existing liver disease. Furthermore, improved care pathways and individualized therapeutic approaches should be tailored for chronic liver disease patients during COVID-19 course.
